# Metal–Organic Framework-Based Fluorinated Carbon for Li Primary Battery

**DOI:** 10.3390/nano16030197

**Published:** 2026-02-02

**Authors:** Hang Xu, Zhihao Gui, Runzhe Wang, Han Yu, Cong Peng, Yu Li, Wei Feng

**Affiliations:** 1 Institute of Advanced Technology and Equipment, Beijing University of Chemical Technology, Beijing 100029, China; xuhang0810@163.com (H.X.); 13628200292@163.com (Z.G.); www1816523188@163.com (R.W.); yh18131430265@163.com (H.Y.); cpeng@buct.edu.cn (C.P.); 2 School of Materials Science and Engineering and Tianjin Key Laboratory of Composite and Functional Materials, Tianjin University, Tianjin 300072, China

**Keywords:** Li/CF_x_ battery, ZIF-8, acid etching, energy density, porous structure

## Abstract

Li/fluorinated carbon (CF_x_) batteries have attracted considerable attention in the field of energy storage owing to their excellent energy density and long storage life. However, the development of CF_x_ cathodes is restricted by their poor conductivity at high degrees of fluorination. Herein, ZIF-8-based fluorinated carbon with a well-developed network structure was fabricated via gas-phase fluorination and acid treatment. Moreover, treatment at a low fluorination temperature of 180 °C for 4 h and acid washing endowed the obtained fluorinated carbon (HFG@ZIF-8) with a high F/C (1.62), favorable specific surface area (207 m^2^ g^−1^), unique porous channels, and highly electrochemically active C–F bonds, resulting in a maximum specific capacity (1143.4 mAh g^−1^) and energy density (2614.8 Wh kg^−1^) at 0.02 C. The superior Li^+^ transport efficiency, with diffusion coefficients ranging from 1.47 × 10^−11^ to 1.93 × 10^−17^ cm^2^ s^−1^, enables HFG@ZIF-8 to deliver 453.4 mAh g^−1^ at 5 C with no voltage delay. Therefore, this work provides an innovative strategy for the preparation of high-performance CF_x_ cathodes.

## 1. Introduction

Compared with traditional Li primary battery systems, such as Li/MnO_2_, Li/SOCl_2_, and Li/SO_2_ [1], Li/fluorinated carbon (CF_x_) batteries exhibit irreplaceable advantages including exceptional energy density (2180 Wh kg^−1^), a wide operating temperature range, and long storage life [2,3]. Consequently, they have gained remarkable attention over the past few decades [4,5,6,7,8]. Nevertheless, Li/CF_x_ batteries still suffer from a few drawbacks arising from the intrinsic characteristics of fluorinated carbon such as severe polarization owing to poor conductivity, resulting in decreased power density and voltage delay [9,10]. To address the aforementioned limitations of CF_x_ cathodes, numerous precursors, including graphene [11,12], nanotubes [13,14], carbon fibers [15], and biomass-derived carbon [16], have been explored for Li/CF_x_ batteries and have achieved considerable progress. Previous studies have reported that the precursor structure plays an essential role in the electrochemical performance of Li/CF_x_ batteries because the formed CF_x_ partially inherits the carbon source structure, including the particle size, pores, and heteroatoms, and demonstrates a significant effect on conductivity and Li^+^ diffusion [17]. On one hand, the porous structure of the carbon source is partially retained by CF_x_, providing channels for Li⁺ transport [18]; on the other hand, heteroatoms regulate the bonding mode of C–F bonds [19,20], thereby affecting conductivity and reaction kinetics. The above results demonstrate the critical role of the precursor in preparing high-performance CF_x_ electrodes.

Recently, metal–organic frameworks (MOFs), composed of organic ligands and metal ions, have attracted substantial interest owing to their adjustable specific surface area, unique multi-dimensional morphology, and favorable electrochemical properties, making them promising for fabricating electrode materials for energy storage [21,22], environmental control [23,24], and biomedical applications [25,26]. Nevertheless, relatively few reports have investigated MOF-based fluorinated carbon for Li/CF_x_ primary batteries. As a subfamily of MOFs, zeolitic imidazolate frameworks (ZIFs) inherit the merits of high specific surface area, structural tunability, and chemical stability. ZIF-8, formed by zinc ions and imidazole ligands, has been regarded as a promising material in various fields [27]. Alex et al. found that ZIF-8 maintained structural stability at temperatures below 300 °C [28]. Li et al. prepared supercapacitor electrodes based on polydopamine/ZIF-8/wood composites and achieved an excellent power density of 1000 mW cm^−2^ [29]. Obodo R. M. et al. used ZIF-8 as a valuable component due to its chemical stability; they interconnected ZIF-8 with Bi_2_O_3_@MoO_3_ to prepare advanced supercapacitor electrodes which exhibited remarkable cyclic stability at a 1.0 A g^−1^ current density [30]. Yang et al. prepared a novel sulfonated metal–organic framework material (ZIF-8-SO_3_H) and applied it to the separator of lithium–sulfur batteries; the ZIF-8-SO_3_H significantly enhanced the cycling stability and rate capability, which was attributed to the superior pore regulation capability of ZIF-8 [31]. Considering the characteristics of ZIF-8, exploring further possibilities in broader application fields is critical.

Herein, ZIF-8-based fluorinated carbon with excellent electrochemical properties was prepared via direct carbonization, gas-phase fluorination, and acid washing processes. Endowed with internal channels provided by a favorable porous network structure after acid etching, the obtained fluorinated carbon (HFG@ZIF-8) exhibits outstanding electrochemical performance, including a specific capacity of 1143 mAh g^−1^ and an energy density of 2614.8 Wh kg^−1^, with no discharge delay even at a high current density of 5 C.

## 2. Materials and Methods

### 2.1. Materials

Methanol (GR) and ethanol (GR) were obtained from Shandong Bluestar New Materials Co., Ltd. (Jinan, China) Zn(NO_3_)_2_·6H_2_O (AR) was purchased from InnoChem Co., Ltd. (Beijing, China) 2-Methylimidazole (98%) was obtained from Shanghai Macklin Biochemical Co., Ltd. (Shanghai, China) Argon gas (99.9%) and F_2_/N_2_ (the purity of fluorine gas is 20%) gas were supplied by Shengtang Gas Co., Ltd. (Tianjin, China). Hydrochloric acid (AR) was purchased from Beijing Tong Guang Fine Chemicals Company. All reagents and solvents were obtained from commercial sources and used directly without further purification. Deionized water (18.2 MΩ cm) was used throughout the preparation process.

### 2.2. Preparation of ZIF-8 and G@ZIF-8

Zn(NO_3_)_2_·6H_2_O (2.97 g, 10 mmol) was dissolved in methanol (200 mL) and denoted as solution A. 2-Methylimidazole (3.28 g and 40 mmol) was dissolved in methanol (200 mL) and denoted as solution B. Solution B was slowly dropped into solution A and stirred for 24 h. The resulting mixture was centrifuged at 6000 r min^−1^ for 5 min and washed several times with methanol. The obtained white product (ZIF-8) was dried at 80 °C overnight. Subsequently, ZIF-8 (2.50 g) was heated to 800 °C for 3 h in a tube furnace under flowing argon at a heating rate of 5 °C min^−1^ and the resulting product was denoted as G@ZIF-8.

### 2.3. Preparation of FG@ZIF-8x and HFG@ZIF-8x

The G@ZIF-8 (200 mg) was placed into a Monel reactor and evacuated using a vacuum. The reactor was then heated to a selected temperature ranging from 100 °C to 220 °C with intervals of approximately 40 °C. Upon reaching the target temperature, the reactor was filled with pure F_2_/N_2_ gas to a pressure of 0.03 MPa, and the fluorination reaction was maintained for 4 h. The resulting fluorinated carbon was denoted as FG@ZIF-8(x), where x represents the reaction temperature. FG@ZIF-8(x) (200 mg) was subsequently added to a mixed solution containing hydrochloric acid (1 mol L^−1^, 40 mL) and ethanol (40 mL) and stirred at 800 r min^−1^ for 6 h, followed by filtration and repeated washing till neutral. The obtained powders were dried at 120 °C overnight and denoted as HFG@ZIF-8(x).

### 2.4. Characterization

The morphology of ZIF-8, G@ZIF-8, FG@ZIF-8, and HFG@ZIF-8 was observed using scanning electron microscopy (SEM, S-4700, Hitachi High-Technologies Corporation, Tokyo, Japan) and transmission electron microscopy (TEM, Tecnai F20, Thermo Fisher Scientific Inc., Waltham, MA, USA), with the scanning voltage set to 10 kV. Fourier transform infrared (FTIR) spectra in the range of 500–4000 cm^−1^ were recorded using an infrared spectrophotometer (Thermo Scientific Nicolet 5700, Thermo Fisher Scientific Inc., Waltham, MA, USA) to identify the functional groups of the prepared materials. X-ray diffraction (XRD) patterns were obtained using an X-ray diffractometer (Ultima IV, Rigaku Corporation, Tokyo, Japan) with Cu Kα radiation (λ = 1.5406 Å, 40 kV, 40 mA) over a scanning range of 5–90°; the samples were washed with ethanol 4 times and dried at 60 °C for 24 h before XRD study. X-ray photoelectron spectroscopy (XPS) was performed using a spectrometer (Thermo Scientific K-Alpha) equipped with an Al anode source operated at 15 kV to analyze the chemical composition of the materials. Raman spectra were recorded using a Horiba LabRAM HR Evolution spectrometer (HORIBA Scientific, Kyoto, Japan). N_2_ adsorption–desorption isotherms were measured using a Micromeritics ASAP 2460 analyzer (Micromeritics Instrument Corporation, Norcross, GA, USA); the degassing temperature and degassing time in the BET test were set at 120 °C and 8 h, respectively, and the specific surface area was calculated using the Brunauer–Emmett–Teller (BET) method.

### 2.5. Electrochemical Tests

The electrochemical performance of HFG@ZIF-8x as the cathode material of Li/CF_x_ batteries in the form of coin cells was evaluated. Specifically, the mixed preparation electrode was prepared with HFG@ZIF-8x (80 wt.%), ketjen black (10 wt.%), and polyvinylidene fluoride (10 wt.%) binder, dispersed in N-methylpyrrolidone (NMP) to form a homogeneous slurry, and then cast on an Al foil and vacuum-dried at 120 °C for 12 h (Figure S1). The average weight load of the active substance on the Al foil was approximately 1–2 mg cm^−2^. A Li/HFG@ZIF-8x battery coin cell (CR2032) was assembled with a Li metal plate as the anode, HFG@ZIF-8x as the cathode, Celgard 2034 membranes as the separators, and 1.0 M LiFSI in propylene carbonate/dimethoxy ethane (PC/DME, 1:1 vol) as the electrolyte (Figure S2). The FG@ZIF-8x cathode was prepared using the same proportions and procedure. The fabricated Li/HFG@ZIF-8x coin cells were discharged at 25 °C at different current densities (Land CT2001A Battery Test System, LAND_7.4.7.6-4, Wuhan Land Electronic Co., Ltd., Wuhan, China), and the discharge-measured termination voltage was 1.5 V. Electrochemical impedance spectroscopy (EIS) was performed on an electrochemical workstation in the frequency range of 0.01 Hz–100 kHz. (CHI 600E, Chenhua Instrument Co., Ltd., Shanghai, China). The galvanostatic intermittent titration technique (GITT) was used to discharge the cell at a current of 0.1 C for 10 min, followed by resting to ensure the voltage reached a steady state (40 min), and discharge and relaxation process was repeated until the voltage of the battery dropped to 1.5 V. Cyclic voltammetry (CV) curves were recorded at different scan rates from 10 to 60 mV s^−1^ to determine the double-layer capacitance. Calculation of the effective active surface area (ECSA) is provided in the Supplementary Materials.

## 3. Results and Discussion

**Figure 1:**
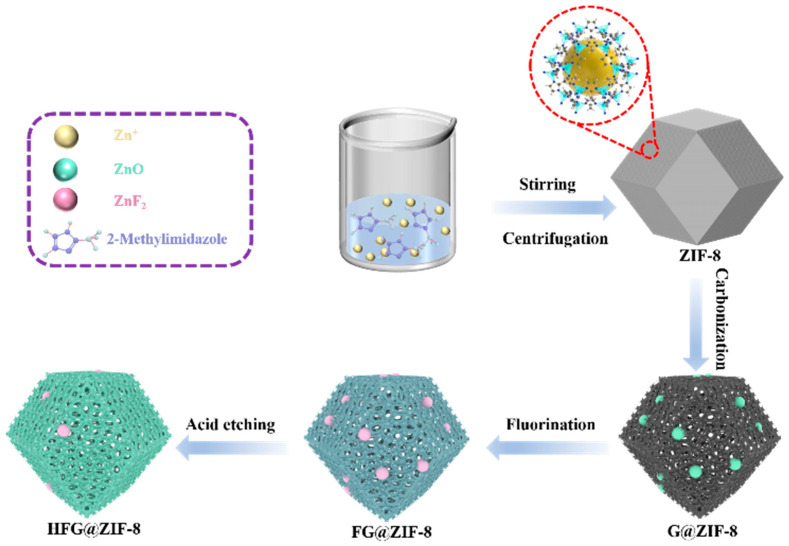
Schematic of fabrication of high-performance HFG@ZIF-8.

**Figure 2:**
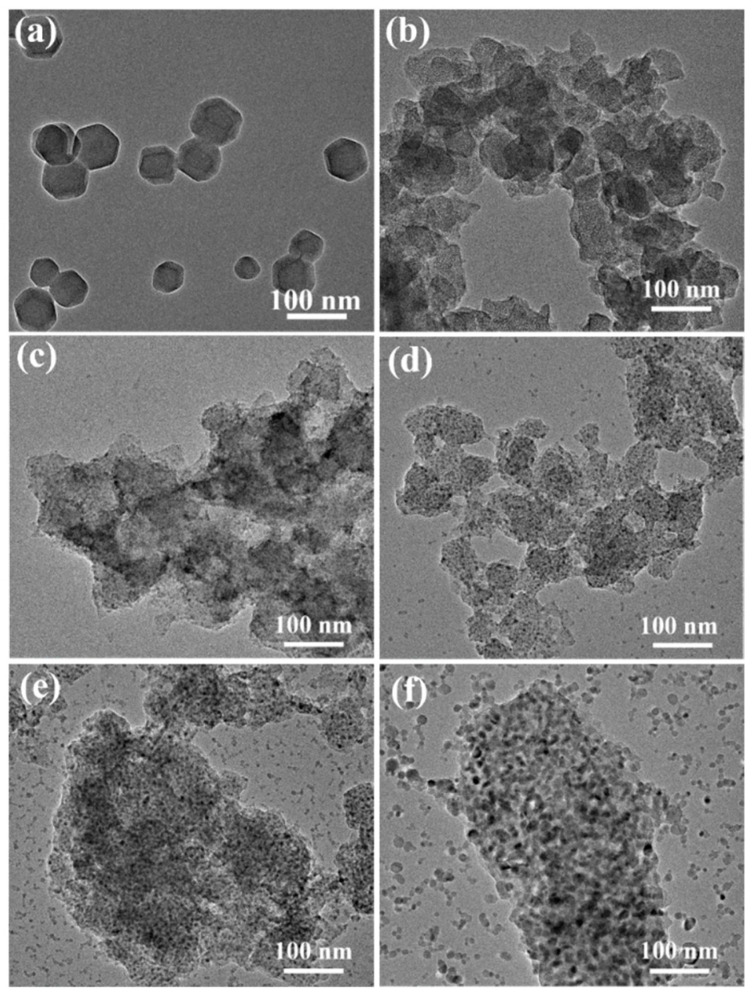
TEM images of ZIF-8 (**a**), G@ZIF-8 (**b**), FG@ZIF-8(100) (**c**), FG@ZIF-8(140) (**d**), FG@ZIF-8(180) (**e**), and FG@ZIF-8(220) (**f**).

The fabrication procedure of HFG@ZIF-8 is illustrated in Figure 1, and the morphology and microstructure of the samples were characterized by SEM and TEM. As shown in Figure 2a and Figure S3a, the synthesized ZIF-8 crystals, which are assembled from Zn^2+^ and 2-methylimidazole via coordination interactions, exhibit a typical rhombic dodecahedral morphology with uniform particle sizes of approximately 50 nm. The samples show smooth surfaces with well-defined edges, consistent with previous reports, further confirming the successful synthesis of ZIF-8 [32,33].

As can be seen in the SEM images of G@ZIF-8 (Figure 2b and Figure S3b), although the particle size is considerably reduced after carbonization and the morphology evolves into a rougher spherical structure, G@ZIF-8 retains a porous network structure, which is favorable for F_2_ diffusion. After fluorination, the samples exhibit a uniform porous structure at 140 °C. However, with continuous etching by F_2_ at higher fluorination temperatures, particles come into contact, become compressed and partially covered by pores, leading to a reduction in pore number and pore size, due to the extremely corrosive nature of F_2_ gas at high temperature. Various porous structures interconnect to form internal channels which serve as efficient supply pathways that accelerate fluorine diffusion and thereby promote low-temperature fluorination (Figure 2c–f and Figure S3c–f). The degree of structural irregularity increases after fluorination, and distinct pore structures can be observed. As the fluorination temperature increases, the content of carbon–fluorine bonds increases accordingly. Meanwhile, the internal ZnO formed during carbonization is gradually transformed into ZnF_2_ crystals under the F_2_ atmosphere. Elemental mapping of G@ZIF-8 and FG@ZIF-8 confirms the uniform distribution of Zn and F, indicating successful ZIF-8 synthesis. It can be observed that the surface of HFG@ZIF-8 exhibits a loose, porous structure after acid etching, indicating the formation of ZnF_2_ and effective fluorination (Figure S4).

After acid etching, the surface of HFG@ZIF-8 exhibits a loose and porous structure, indicating that the formed ZnF_2_ is successfully removed and that additional Li^+^ transport pathways are generated (Figure S5). Moreover, with increasing fluorination temperature, the structure of HFG@ZIF-8 becomes progressively damaged after acid washing, resulting in smaller particle sizes and indicating a higher degree of acid etching. Owing to the formation and subsequent precipitation of ZnF_2_ from the surface, corresponding ZnF_2_ attachment sites are created. Consequently, the structure of HFG@ZIF-8 partially collapses owing to the loss of ZnF_2_ crystal support. In addition, acid treatment not only increases the specific surface area of HFG@ZIF-8, thereby enhancing electrolyte contact and improving electrochemical reaction efficiency, but also exposes internal nanoscale pores and low-fluorination-degree CF_x_ regions through the newly formed cross-sections [34]. This provides additional Li^+^ transport pathways, shortens the Li^+^ diffusion distance, and ultimately further optimizes the electrochemical performance of Li/CF_x_ batteries. Compared with FG@ZIF-8, elemental mapping of HFG@ZIF-8 shows that a portion of ZnF_2_ is successfully removed after acid etching (Figure S6). According to the high-resolution TEM (HRTEM) results (Figure S7), ZIF-8 and FG@ZIF-8 exhibit typical amorphous structures. The ZnO crystals formed during carbonization may be encapsulated by amorphous carbon. In addition, two interplanar spacings of 0.280 and 0.331 nm are observed, corresponding to the (100) plane of ZnO and the (110) plane of ZnF_2_ [35,36,37], respectively, which is consistent with the selected area electron diffraction results. Fluorine atoms penetrate the internal carbon network through the pores and precipitate on the surface as the temperature increases, leading to an increased ZnF_2_ content. TEM and HRTEM analyses (Figures S8 and S9) confirm that ZnF_2_ is effectively removed after acid washing. However, residual ZnF_2_ is still detected in HFG@ZIF-8(220), which is attributed to the high ZnF_2_ content that limits the efficiency of acid washing.

**Figure 3:**
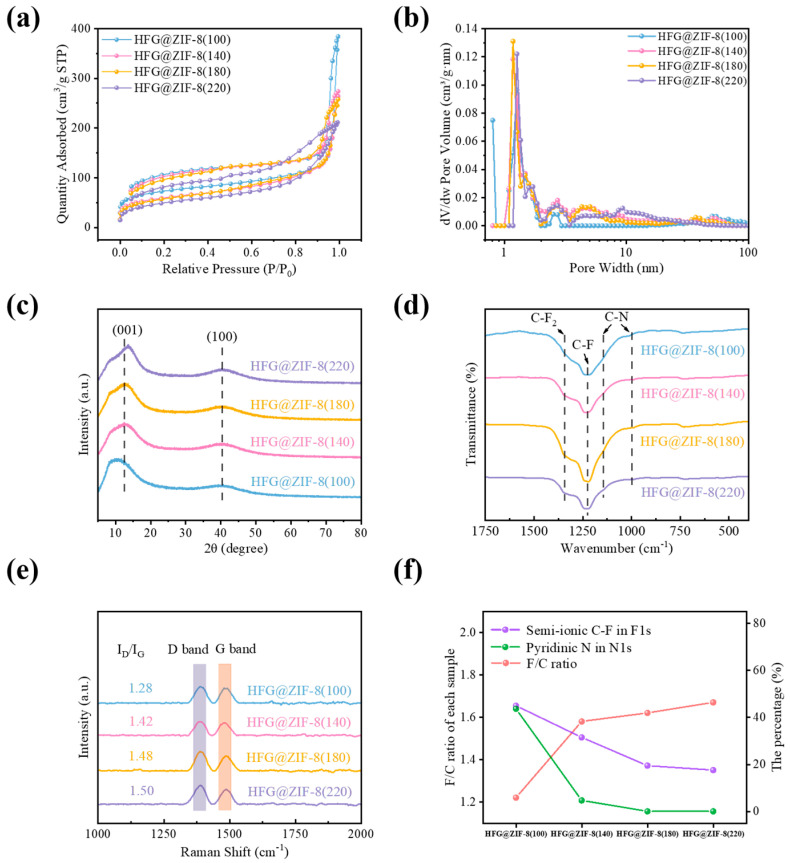
The N_2_ isothermal adsorption–desorption curves (**a**) and the pore size distribution (**b**) of HFG@ZIF-8; the XRD patterns (**c**), FT-IR spectra (**d**), and Raman spectra of HFG@ZIF-8 (**e**); the F/C ratio and the percentage of semi-ionic C–F in F 1s and of pyridinic N in N 1s as a function of fluorination temperature of HFG@ZIF-8 (**f**).

Nitrogen adsorption–desorption isotherm analysis was conducted to investigate the specific surface area and pore size distribution of the samples. ZIF-8 exhibits a typical type I isotherm with an H1 hysteresis loop (Figure S10a), indicating a microporous structure [38]. In contrast, G@ZIF-8, FG@ZIF-8(100), FG@ZIF-8(140), and FG@ZIF-8(180) exhibit type IV isotherms with evident hysteresis loops, suggesting predominantly mesoporous and macroporous structures (Figure S10b). The specific surface areas of G@ZIF-8, FG@ZIF-8(100), FG@ZIF-8(140), FG@ZIF-8(180), and FG@ZIF-8(220) are 200.3, 323.9, 194.3, 171.2, and 5.2 m^2^ g^−1^, respectively. With increasing fluorination temperature, the adsorption capacity of FG@ZIF-8 gradually decreases, indicating the disappearance of pore structures [39], which is consistent with the SEM observations. After acid washing, HFG@ZIF-8 still exhibits type IV isotherms (Figure 3a), indicating the presence of mesopores and macropores [40], and the specific surface areas are 266.7, 215.5, 207.5, and 179.3 m^2^ g^−1^, respectively. Regarding pore size distribution, the number of micropores decreases due to structural collapse after carbonization. As the fluorination temperature increases, the fluorination reaction further etches the material surface, leading to a reduction in specific surface area. In addition, the pore size distribution of HFG@ZIF-8 still demonstrates a hierarchical porous structure, indicating that the framework is preserved after acid treatment. It should be noted that the removal of ZnF_2_ generates abundant micropores and mesopores, which is favorable for electrochemical performance (Figure S10c,d and Figure 3b). The detailed data are summarized in Tables S1 and S2.

As shown in Figure S11a, ZIF-8 exhibits a typical XRD pattern with diffraction peaks at 7.2°, 10.3°, 12.7°, 14.5°, 16.4°, and 18.0°, corresponding to the (011), (002), (112), (022), (013), and (222) planes, respectively, confirming the successful synthesis of ZIF-8 [41,42]. After carbonization, new diffraction peaks appear at 26.2° and 43.7°, which are attributed to the (002) and (101) planes (Figure S11b). Two broad peaks located at 13° and 41° originate from the (001) and (100) planes of CF_x_ [43]. In addition, the diffraction peaks at 27.5°, 34.8°, and 52.9° in FG@ZIF-8 correspond to the (110), (101), and (211) planes of ZnF_2_ (Figure S11c). The sharpening of XRD signals indicates that ZnO is gradually converted into ZnF_2_ as the fluorinated temperature increases. The disappearance of the characteristic ZnF_2_ peaks after acid washing confirms the effectiveness of the treatment. Furthermore, the 2*θ* values of the (001) diffraction peaks and the corresponding interlayer distances of HFG@ZIF-8 in their XRD patterns are shown in Table S3; the (001) peak shifts toward a higher angle, indicating reduced interlayer spacing in HFG@ZIF-8, which is attributed to the removal of ZnF_2_ crystals (Figure 3c). In addition, the particle size (15–17 nm) calculated from XRD via the Scherrer equation is consistent with the results of SEM and TEM.

As can be seen in the FTIR spectra of ZIF-8 and G@ZIF-8 (Figure S12), the peaks at 990, 1145, and 1584 cm^−1^ correspond to C–N and C=N stretching vibrations. The Zn–N stretching vibration at 422 cm^−1^ confirms the successful synthesis of ZIF-8. After carbonization, the intensity of C–N bonds decreases, the C–H stretching vibrations at 2930 and 3140 cm^−1^ disappear, and the Zn–N vibration peak is significantly weakened, indicating the cleavage of C–N and Zn–N bonds and the formation of O–C=O bonds and ZnO [44]. The emergence of new peaks in the range of 1150–1212 cm^−1^ further indicates the formation of fluorine-containing groups after fluorination. Moreover, the peak at 450 cm^−1^ observed in FG@ZIF-8 confirms the formation of ZnF_2_, which disappears after acid washing (Figure 3d).

As shown in the Raman spectra of G@ZIF-8, FG@ZIF-8, and HFG@ZIF-8 (Figure S13 and Figure 3e), two characteristic peaks are observed at approximately 1340 and 1585 cm^−1^, corresponding to the D band and G band [45], respectively. The I_D_/I_G_ ratio increases with increasing fluorination temperature. On one hand, the carbon skeleton is etched by highly reactive F_2_, breaking C–C bonds and compromising the structural integrity. On the other hand, the formation of ZnF_2_ crystals further aggravates the degree of structural disorder, leading to an increased I_D_/I_G_ value. After acid washing, HFG@ZIF-8(220) reaches a maximum I_D_/I_G_ value of approximately 1.5, which can be attributed to the removal of ZnF_2_ and the consequent enhancement of structural disorder in HFG@ZIF-8.

XPS was employed to investigate the evolution of functional groups in ZIF-8, G@ZIF-8, FG@ZIF-8, and HFG@ZIF-8. Compared with FG@ZIF-8, the absence of Zn signals in HFG@ZIF-8 further confirms the effective removal of ZnF_2_ through acid treatment, which is consistent with previous results (Figures S14 and S15a,b). The calculated F/C ratio of FG@ZIF-8 increases with fluorination temperature and reaches a maximum value of 1.47 at 220 °C (Figure S15c). In addition, the F/C ratio of HFG@ZIF-8 is higher than that of FG@ZIF-8, which may be attributed to structural collapse after acid washing, exposing internal fluorine-containing groups (Figure S15d). The composition contents of ZIF-8, G@ZIF-8, FG@ZIF-8 and HFG@ZIF-8 from the XPS spectra are summarized in Tables S4 and S5.

The high-resolution N 1s spectra can be deconvoluted into three peaks located at 398, 399, and 399.8 eV, corresponding to pyrrolic N, Zn–N, and pyridinic N, respectively. The characteristic peaks at 1021.95 and 1044.89 eV correspond to the Zn 2p3/2 and Zn 2p1/2 signals (Figure S16). With increasing fluorination temperature, the content of semi-ionic C–F bonds decreases due to their conversion into covalent C–F bonds (Figure S17a). The emergence of a Zn–F peak at 685.7 eV indicates the transformation of ZnO into ZnF_2_ (Figure S17b). Compared with FG@ZIF-8, HFG@ZIF-8 exhibits a higher content of semi-ionic C–F bonds, which may be attributed to structural collapse after acid treatment, exposing internal low-fluorination-degree C–F bonds (Figure S18a). Moreover, the abundance of highly electrochemically active functional groups is beneficial for the electrochemical performance of Li/HFG@ZIF-8 batteries. As the fluorination temperature increases, the content of semi-ionic C–F bonds decreases, whereas the contents of covalent C–F bonds, –CF_2_, and –CF_3_ increase (Figure S18b), consistent with the trend observed for FG@ZIF-8. Based on the N 1s spectra of FG@ZIF-8, the content of pyridinic N decreases with increasing fluorination temperature (Figures S17c and S18c). This behavior may be attributed to the preferential fluorination at nanopore edges, where pyridinic N and pyrrolic N are predominantly distributed, leading to the detachment of nitrogen-containing groups from the carbon framework [46]. The variation in the F/C ratio and in the proportion of semi-ionic C–F bonds and pyridinic N as the fluorination temperature of fluorinated products changes is illustrated in Figure S19 and Figure 3f. The detailed peak assignments and relative proportions are summarized in Tables S6–S9.

**Figure 4:**
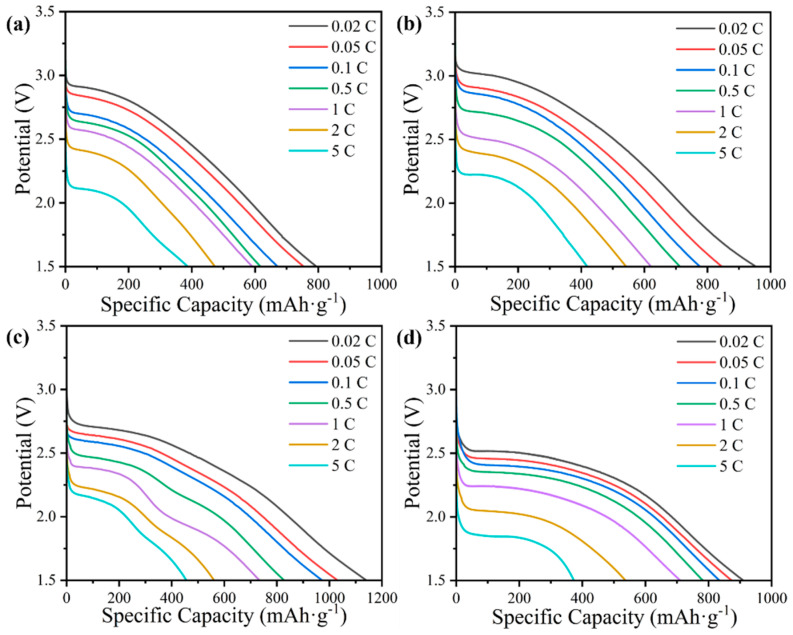
Galvanostatic discharge curves of (**a**) HFG@ZIF-8(100), (**b**) HFG@ZIF-8(140), (**c**) HFG@ZIF-8(180), and (**d**) HFG@ZIF-8(220).

FG@ZIF-8 samples were used as cathode materials for Li/CF_x_ batteries, and their electrochemical performances were evaluated. The galvanostatic discharge curves at different current densities with a cut-off voltage of 1.5 V are presented in Figure S20. The fluorination temperature is beneficial for increasing specific capacity but detrimental to the discharge plateau owing to the formation of excessive perfluorinated groups (–CF_2_ and –CF_3_), as confirmed by the XPS results [47]. The specific capacities of FG@ZIF-8(100), FG@ZIF-8(140), FG@ZIF-8(180), and FG@ZIF-8(220) discharged at 0.02 C are 597.6, 742.3, 982.5, and 765.4 mAh g^−1^, respectively, with corresponding energy densities of 1349.6, 1757.5, 1445.3, and 1273.7 Wh kg^−1^. It should be noted that the specific capacity of FG@ZIF-8 decreases when the fluorination temperature exceeds 220 °C. This behavior can be attributed to the increased particle size, which covers the surface pore structure of FG@ZIF-8(220), thereby reducing the specific surface area and pore volume, as indicated by the SEM images and BET results. This hinders effective contact between CF_x_ and the electrolyte and is ultimately unfavorable for battery capacity [48]. The galvanostatic discharge performances of HFG@ZIF-8 cathodes were also evaluated (Figure 4). The specific capacities of HFG@ZIF-8(100), HFG@ZIF-8(140), HFG@ZIF-8(180), and HFG@ZIF-8(220) are 795.5, 953.0, 1143.4, and 912.0 mAh g^−1^ at 0.02 C, respectively, with corresponding energy densities of 1883.1, 2328.2, 2614.8, and 2026.8 Wh kg^−1^. These values are higher than those of commercial fluorinated graphite (GF) (Figure S18e). Furthermore, the HFG@ZIF-8 cathodes demonstrate superior rate performance, delivering specific capacities of 388.2, 409.8, 453.4, and 378.1 mAh g^−1^ at 5 C without voltage delay. The nanopores generated by the removal of ZnF_2_ during acid etching provide additional transport pathways for Li^+^ diffusion and sufficient space to accommodate low-conductivity LiF discharge products. In addition, the etching effect of acid washing increases the specific surface area and shortens the Li^+^ diffusion path, thereby improving electrochemical reaction efficiency. Furthermore, the exposure of low-fluorination-degree CF_x_ after acid treatment increases the proportion of highly active semi-ionic C–F bonds in the HFG@ZIF-8 cathode due to structural collapse, further facilitating Li^+^ diffusion.

The GITT was an effective method used to determine the Li^+^ diffusion coefficient in the batteries [49,50]. In particular, FG@ZIF-8 and HFG@ZIF-8 cathodes were assembled into coin cells, as shown in Figure S21. The Li^+^ diffusion coefficient (D_Li_^+^) of FG@ZIF-8 ranges from 9.68 × 10^−11^ to 3.31 × 10^−15^ cm^2^ s^−1^ and exhibits a sharp increase during the initial discharge stage, followed by a gradual decrease as discharge proceeds. Conductive carbon generated during the initial discharge enhances the conductivity of the cathode material, resulting in an increased D_Li_^+^. However, the formation of LiF crystals as the reaction progresses impedes Li^+^ migration, resulting in a decline in D_Li_^+^. The D_Li_^+^ values of HFG@ZIF-8 range from 1.47 × 10^−11^ to 1.93 × 10^−17^ cm^2^ s^−1^ and are higher than those of GF (Figure S22), indicating superior Li^+^ transport efficiency. Moreover, the D_Li_^+^ values of HFG@ZIF-8 are significantly enhanced compared with those of FG@ZIF-8. The newly formed nanopores generated by ZnF_2_ removal provide additional pathways for Li^+^ migration, while the increased specific surface area improves electrolyte contact and shortens Li^+^ diffusion distances, ultimately resulting in a substantial improvement in D_Li_^+^.

Subsequently, EIS measurements were employed to elucidate the underlying causes of the electrochemical performance differences among the samples [51]. As shown in Figure S23, at open-circuit voltage, the EIS spectra of GF, FG@ZIF-8, and HFG@ZIF-8 comprise a high-frequency semicircle and a low-frequency sloping line, which can be well fitted using the equivalent circuit shown in the inset of Figure S23. The fitted bulk ohmic resistance (R_s_) and charge transfer resistance (R_ct_) values are summarized in Figure S24. Notably, the R_ct_ values are comparable to those obtained in our previous work and increase with rising fluorination temperature for both FG@ZIF-8 and HFG@ZIF-8, which is attributed to the formation of fluorinated species with lower electrochemical activity [43,52]. This trend is consistent with the GITT results. In addition, HFG@ZIF-8 exhibits a lower R_ct_ than FG@ZIF-8 and GF under the same reaction condition, indicating that the surface pore structure formed after acid washing enhances Li^+^ diffusion efficiency and provides more pathways for Li^+^ diffusion, which is favorable for the electrochemical performance of an Li/HFG@ZIF-8 primary battery. It can be observed that the surface of the discharged HFG@ZIF-8(180) cathode is covered with LiF crystals, which are the reaction products (Figure S25). Owing to the poor electrical conductivity of LiF crystals, the charge transfer resistance (R_ct_) increases gradually with the deepening of discharge depth (Figure S26), Moreover, the value of the ECSA of HFG@ZIF-8(180) calculated according to the CV curves is 227 cm^2^, which is consistent with the specific surface area value measured previously. Benefiting from the favorable porous structure after acid treatment, most of the active sites of HFG@ZIF-8(180) are involved in the electrochemical reaction, which explains the excellent electrochemical performance.

## 4. Conclusions

A series of ZIF-8-based fluorinated carbon cathodes with excellent electrochemical performance were synthesized via gas-phase fluorination and acid treatment. Morphological analysis reveals that HFG@ZIF-8 possesses well-developed internal channels, uniformly distributed throughout the material, which facilitate Li^+^ migration. The assembled Li/HFG@ZIF-8 battery delivers a high specific capacity of 1143.4 mAh g^−1^, corresponding to an energy density of 2614.8 Wh kg^−1^ at 0.02 C. The Li^+^ diffusion coefficient is on the order of 10^−13^ cm^2^ s^−1^, indicating favorable electrochemical kinetics, and a high specific capacity of 453.4 mAh g^−1^ is achieved at 5 C. This study provides a novel strategy for the fabrication of high-performance MOF-based fluorinated carbon cathodes for Li primary batteries.

## Data Availability

The original contributions presented in this study are included in the article/Supplementary Material. Further inquiries can be directed to the corresponding authors.

## Supplementary Materials

The following supporting information can be downloaded at https://www.mdpi.com/article/10.3390/nano16030197/s1, Figure S1: The procedure of preparing the HFG@ZIF-8 fluorinated carbon cathodes; Figure S2: The components of HFG@ZIF-8 coin cells; Figure S3: SEM images of ZIF-8 (a), G@ZIF-8 (b), FG@ZIF-8(100) (c), FG@ZIF-8(140) (d), FG@ZIF-8(180) (e), and FG@ZIF-8(220) (f). The inset images show partially enlarged SEM images with a scale bar of 200 nm; Figure S4: SEM images with corresponding elemental mapping of G@ZIF-8 (a) and FG@ZIF-8 (b); Figure S5: SEM images of HFG@ZIF-8(100) (a), HFG@ZIF-8(140) (b), HFG@ZIF-8(180) (c), and HFG@ZIF-8(220) (d). The inset images show partially enlarged SEM images with a scale bar of 200 nm; Figure S6: SEM image with corresponding elemental mapping of HFG@ZIF-8(100); Figure S7: HRTEM images of ZIF-8 (a), G@ZIF-8 (b), FG@ZIF-8(100) (c), FG@ZIF-8(140) (d), FG@ZIF-8(180) (e), and FG@ZIF-8(220) (f). The inset images show the corresponding SAED patterns with a scale bar of 5 nm^−1^; Figure S8: TEM images of HFG@ZIF-8(100) (a), HFG@ZIF-8(140) (b), HFG@ZIF-8(180) (c), and HFG@ZIF-8(220) (d); Figure S9: HRTEM images of HFG@ZIF-8(100) (a), HFG@ZIF-8(140) (b), HFG@ZIF-8(180) (c), and HFG@ZIF-8(220) (d). The inset image shows the corresponding SAED pattern with a scale bar of 5 nm^−1^; Figure S10: The N_2_ isothermal adsorption–desorption curves of ZIF-8, G@ZIF-8, and FG@ZIF-8 (a,b), and the pore size distribution (c,d); Figure S11: XRD patterns of (a) ZIF-8, (b) G@ZIF-8, and (c) FG@ZIF-8; Figure S12: FT-IR spectra of ZIF-8, G@ZIF-8 and FG@ZIF-8; Figure S13: Raman spectra of G@ZIF-8 and FG@ZIF-8; Figure S14: XPS spectra of (a) ZIF-8 and G@ZIF-8, and (b) FG@ZIF-8 and HFG@ZIF-8; Figure S15: XPS spectra of FG@ZIF-8 (a) and HFG@ZIF-8 (b), and the corresponding F/C ratios of FG@ZIF-8 (c) and HFG@ZIF-8 (d); Figure S16: XPS spectra of ZIF-8: (a) C 1s, (b) N 1s, and (c) Zn 2p; XPS spectra of G@ZIF-8: (d) C 1s, (e) N 1s, and (f) Zn 2p; Figure S17: XPS spectra of FG@ZIF-8 prepared at different temperatures: (a) C 1s, (b) F 1s, and (c) N 1s; Figure S18: XPS spectra of HFG@ZIF-8 prepared at different temperatures: (a) C 1s, (b) F 1s, and (c) N 1s; Figure S19: F/C ratio, semi-ionic C–F content in F 1s, and pyridinic N content in N 1s as a function of fluorination temperature for FG@ZIF-8; Figure S20: Galvanostatic discharge curves of (a) FG@ZIF-8(100), (b) FG@ZIF-8(140), (c) FG@ZIF-8(180), (d) FG@ZIF-8(220), and (e) GF; Figure S21: (a) GITT curves of FG@ZIF-8 at 0.1 C, and (b) relationship between D_Li_^+^ and voltage for FG@ZIF-8; Figure S22: (a) GITT curves of HFG@ZIF-8 at 0.1 C, and (b) relationship between D_Li_^+^ and voltage for HFG@ZIF-8; Figure S23: The open-circuit voltage of FG@ZIF-8 (a) and HFG@ZIF-8 (b); the EIS spectra of FG@ZIF-8 (c) and HFG@ZIF-8 (d); Figure S24: Bulk ohmic resistance (R_s_) and charge transfer resistance (R_ct_) of FG@ZIF-8 (a,b) and HFG@ZIF-8 (c,d); Figure S25: SEM images of the HFG@ZIF-8(180) cathode before discharge (a) and after discharge (b,c); Figure S26: EIS spectra of HFG@ZIF-8(180) at different depths of discharge; Figure S27: (a) CV curves of HFG@ZIF-8(180) at different scan rates; (b) linear plots of scan rates versus current density of HFG@ZIF-8(180); Table S1: BET surface area and pore volume of ZIF-8, G@ZIF-8, and FG@ZIF-8; Table S2: BET surface area and pore volume of HFG@ZIF-8; Table S3: The interlayer space of HFG@ZIF-8 (obtained from XRD results); Table S4: Composition contents of ZIF-8, G@ZIF-8, and FG@ZIF-8 derived from XPS spectra; Table S5: Composition contents of HFG@ZIF-8 derived from XPS spectra; Table S6: C 1s peak assignments and relative proportions of ZIF-8, G@ZIF-8, and FG@ZIF-8 derived from XPS spectra; Table S7: F 1s peak assignments and relative proportions of FG@ZIF-8 derived from XPS spectra; Table S8: C 1s peak assignments and relative proportions of HFG@ZIF-8 derived from XPS spectra; Table S9: F 1s peak assignments and relative proportions of HFG@ZIF-8 derived from XPS spectra. Ref. [53] is cited in the Supplementary Material File.

## Abbreviations

The following abbreviations are used in this manuscript:

EIS	electrochemical impedance spectroscopy
GITT	galvanostatic intermittent titration technique
SEM	scanning electron microscopy
TEM	transmission electron microscopy
XPS	X-ray photoelectron spectroscopy
XRD	X-ray diffraction
ZIF	zeolitic imidazolate framework
CF_x_	fluorinated carbon
MOFs	metal–organic frameworks
FTIR	Fourier transform infrared
BET	Brunauer–Emmett–Teller
HRTEM	high-resolution TEM
CV	cyclic voltammetry
ECSA	electrochemical active surface area
