# Supplementary material for: Metal–Organic Framework-Based Fluorinated Carbon for Li Primary Battery
Source: Nanomaterials (Basel). 2026 Feb 2;16(3):197. doi: 10.3390/nano16030197 (PMC12899712; doi:10.3390/nano16030197)
Supplement: Supplementary file 1 [file nanomaterials-16-00197-s001.zip › nanomaterials-4126063-supplementary.pdf]

Article

Metal–Organic Framework-Based Fluorinated Carbon for Li Primary Battery

Hang Xu [1], Zhihao Gui [1], Runzhe Wang [1], Han Yu [1], Cong Peng [1], Yu Li [1,*] and Wei Feng [1,2,*]

1 Institute of Advanced Technology and Equipment, Beijing University of Chemical Technology, Beijing 100029, China; xuhang0810@163.com (H.X.); 13628200292@163.com (Z.G.); www1816523188@163.com (R.W.); yh18131430265@163.com (H.Y.); cpeng@buct.edu.cn (C.P.)

2 School of Materials Science and Engineering and Tianjin Key Laboratory of Composite and Functional Materials, Tianjin University, Tianjin 300072, China

* Correspondence: 2022500016@buct.edu.cn (Y.L.); weifeng@tju.edu.cn (W.F.)

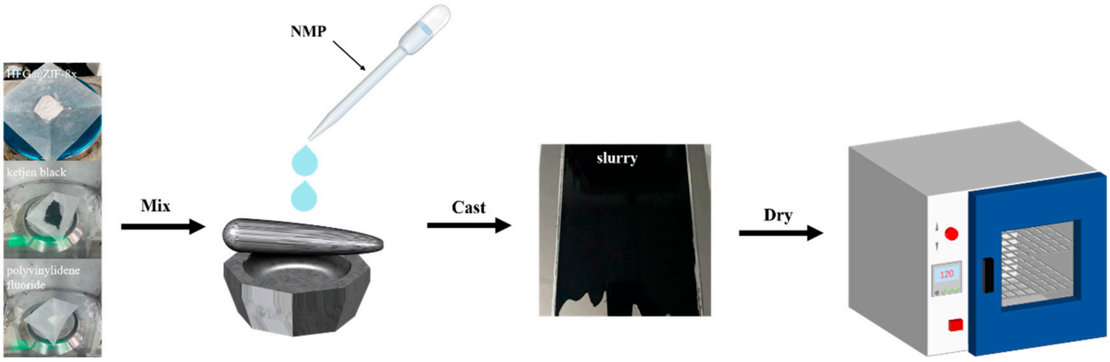

Figure S1 The procedure of preparing the HFG@ZIF-8 fluorinated carbon cathodes.

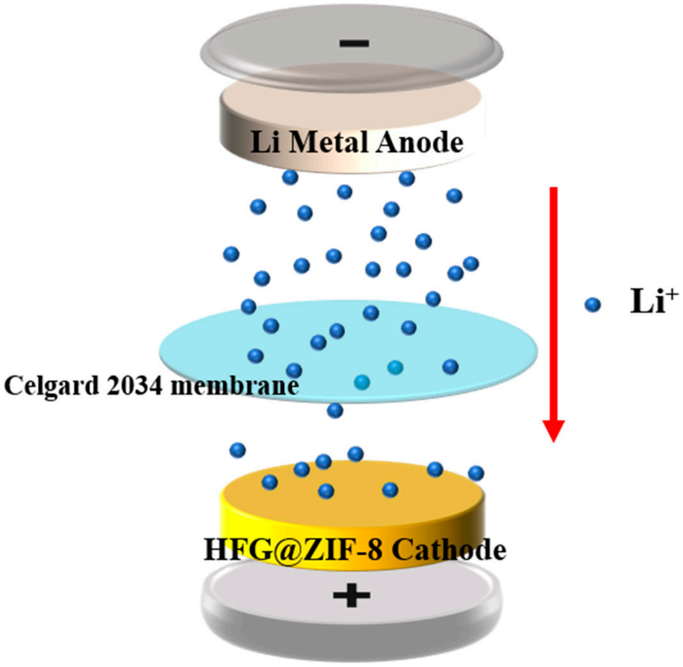

Figure S2 The components of HFG@ZIF-8 coin cells.

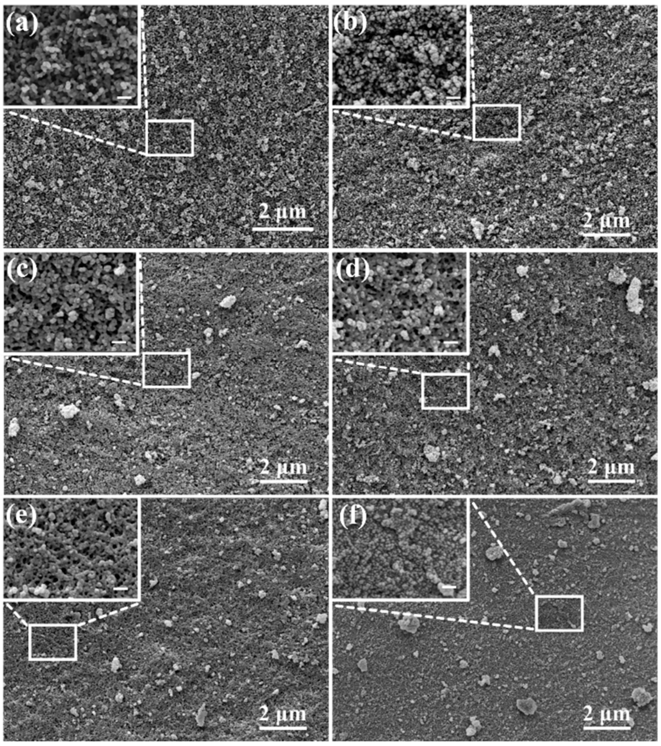

Figure S3 SEM images of ZIF-8 (a), G@ZIF-8 (b), FG@ZIF-8(100) (c), FG@ZIF-8(140) (d), FG@ZIF-8(180) (e), and FG@ZIF-8(220) (f). The inset images show partially enlarged SEM images with a scale bar of 200 nm.

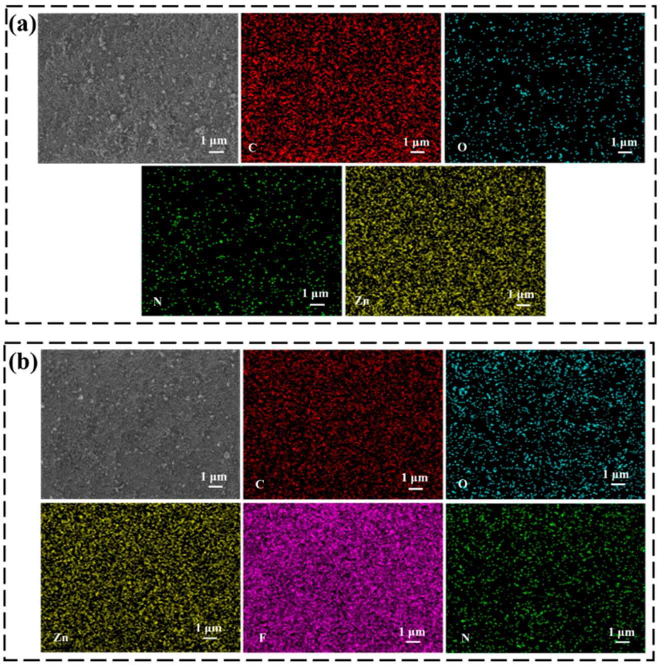

Figure S4 SEM images with corresponding elemental mapping of G@ZIF-8 (a) and FG@ZIF-8 (b).

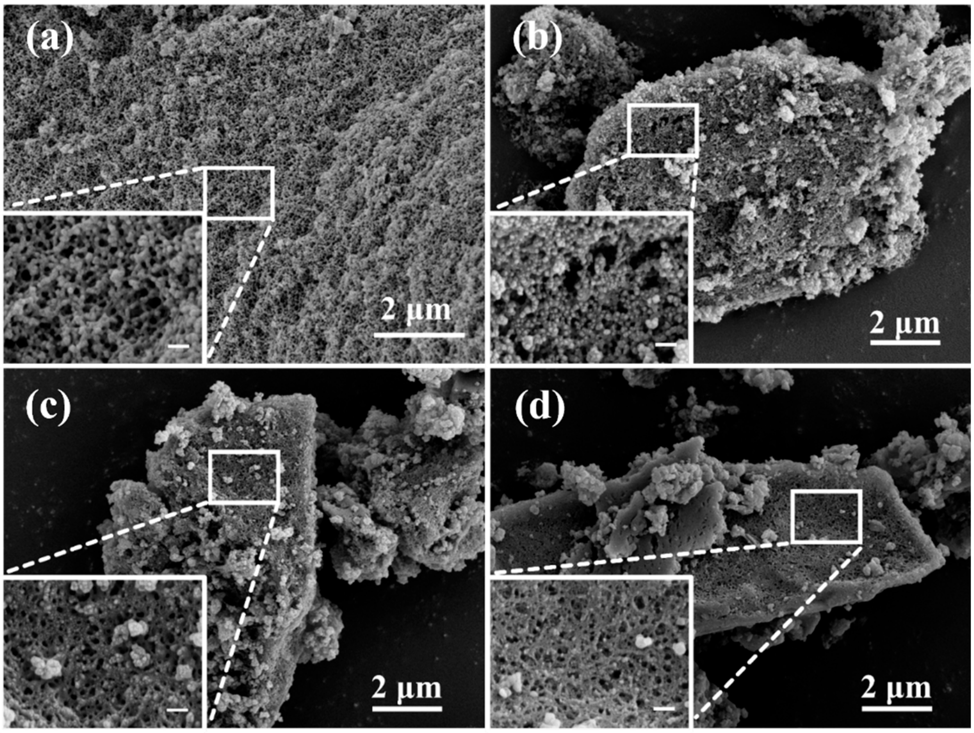

Figure S5 SEM images of HFG@ZIF-8(100) (a), HFG@ZIF-8(140) (b), HFG@ZIF-8(180) (c), and HFG@ZIF-8(220) (d). The inset images show partially enlarged SEM images with a scale bar of 200 nm.

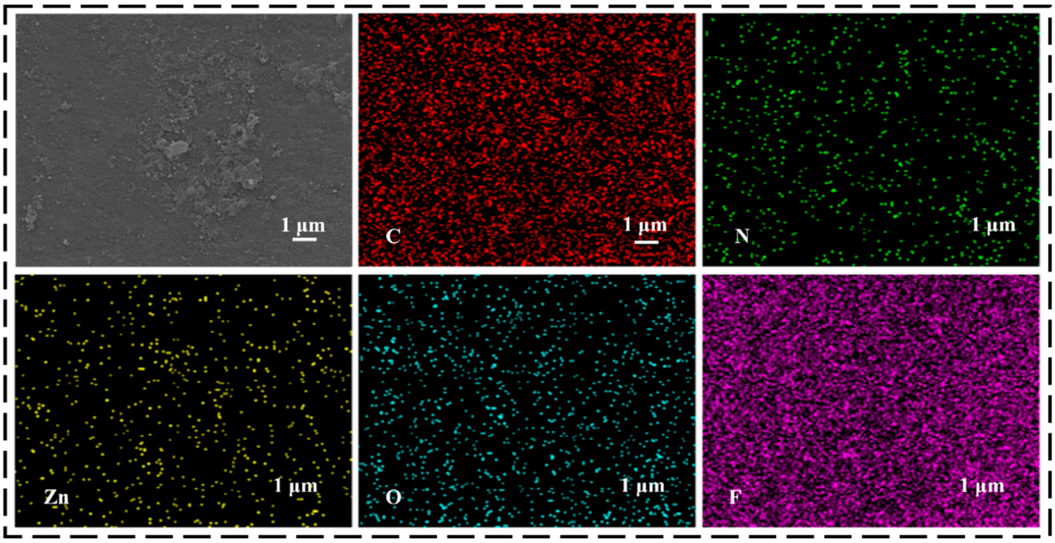

Figure S6 SEM image with corresponding elemental mapping of HFG@ZIF-8(100).

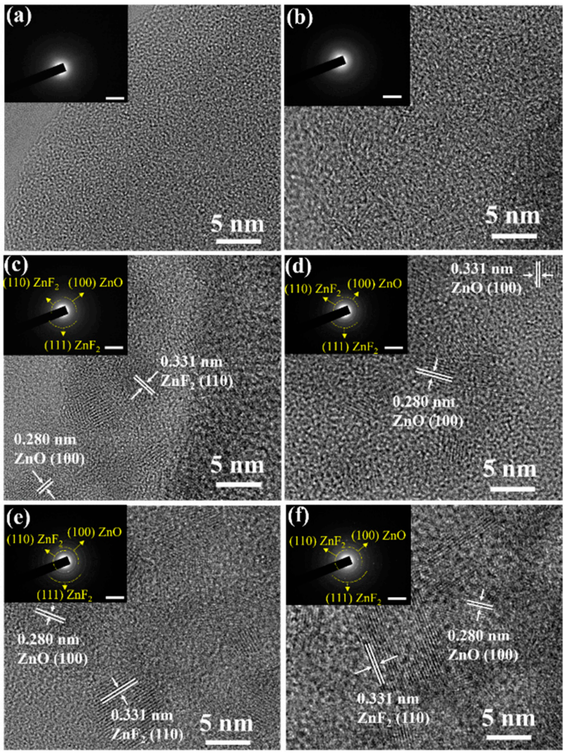

Figure S7 HRTEM images of ZIF-8 (a), G@ZIF-8 (b), FG@ZIF-8(100) (c), FG@ZIF-8(140) (d), FG@ZIF-8(180) (e), and FG@ZIF-8(220) (f). The inset images show the corresponding SAED patterns with a scale bar of 5 nm^{-1}.

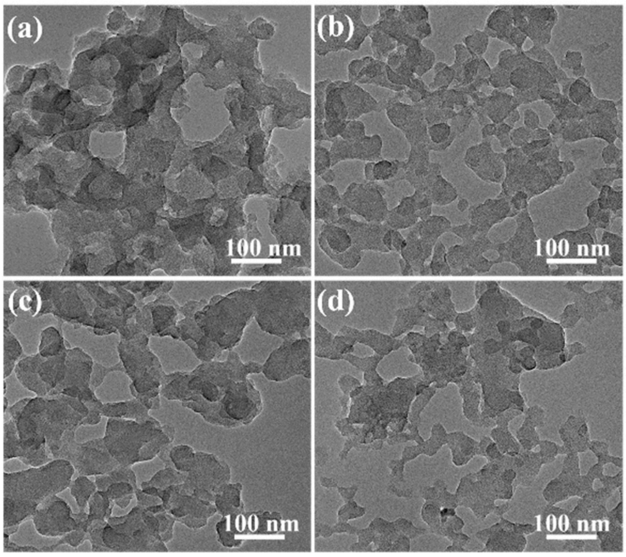

Figure S8 TEM images of HFG@ZIF-8(100) (a), HFG@ZIF-8(140) (b), HFG@ZIF-8(180) (c), and HFG@ZIF-8(220) (d).

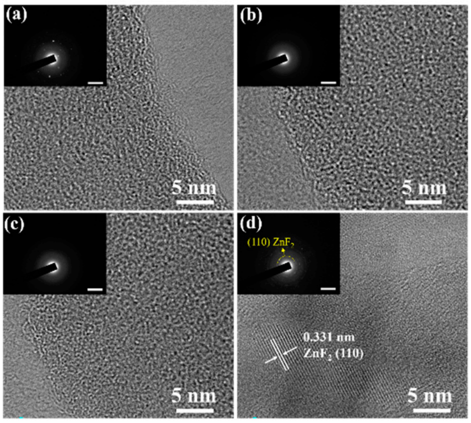

Figure S9 HRTEM images of HFG@ZIF-8(100) (a), HFG@ZIF-8(140) (b), HFG@ZIF-8(180) (c), and HFG@ZIF-8(220) (d). The inset image shows the corresponding SAED pattern with a scale bar of 5 nm⁻¹.

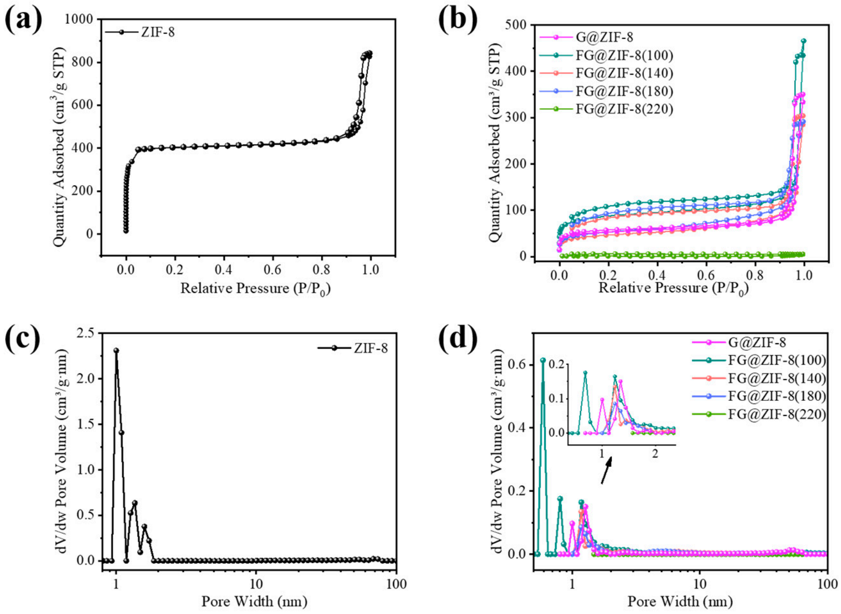

Figure S10 The N_2 isothermal adsorption-desorption curves of ZIF-8, G@ZIF-8, and FG@ZIF-8 (a-b), and the Pore size distribution (c-d).

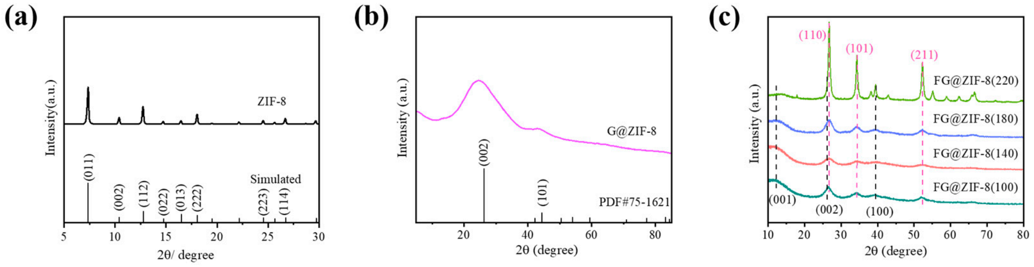

Figure S11 XRD patterns of (a) ZIF-8, (b) G@ZIF-8, (c) FG@ZIF-8.

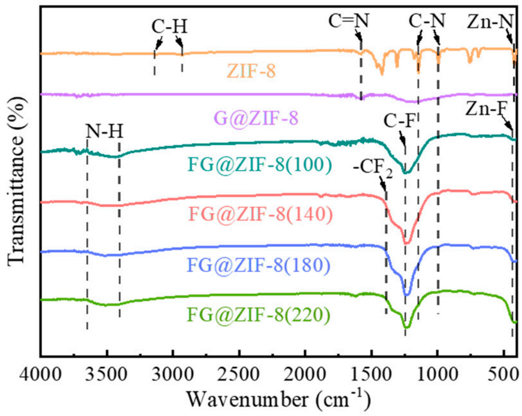

Figure S12 FT-IR spectra of ZIF-8, G@ZIF-8 and FG@ZIF-8.

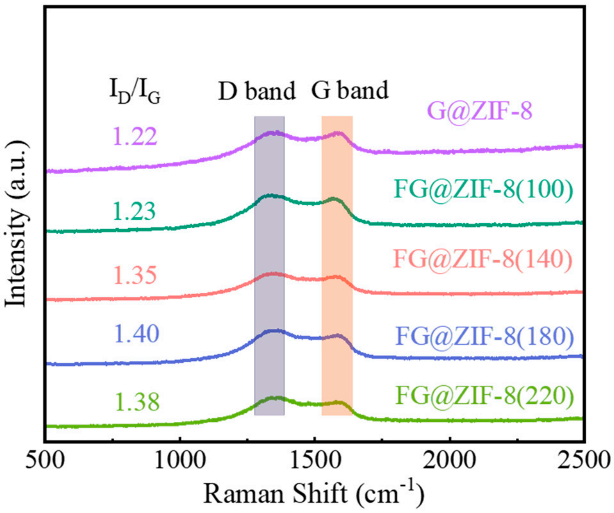

Figure S13 Raman spectra of G@ZIF-8 and FG@ZIF-8.

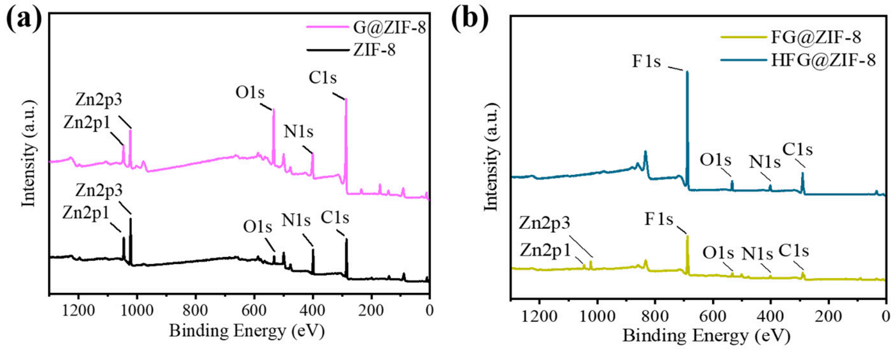

Figure S14 XPS spectra of (a) ZIF-8 and G@ZIF-8, and (b) FG@ZIF-8 and HFG@ZIF-8.

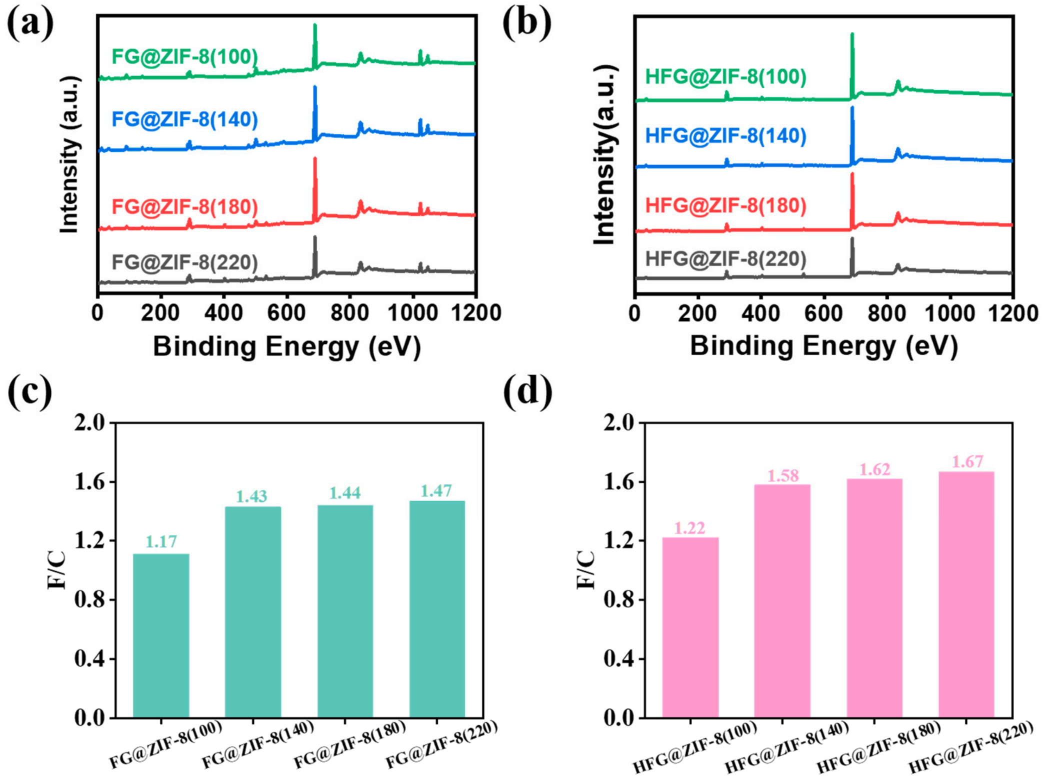

Figure S15 XPS spectra of FG@ZIF-8 (a) and HFG@ZIF-8 (b), and the corresponding F/C ratios of FG@ZIF-8 (c) and HFG@ZIF-8 (d).

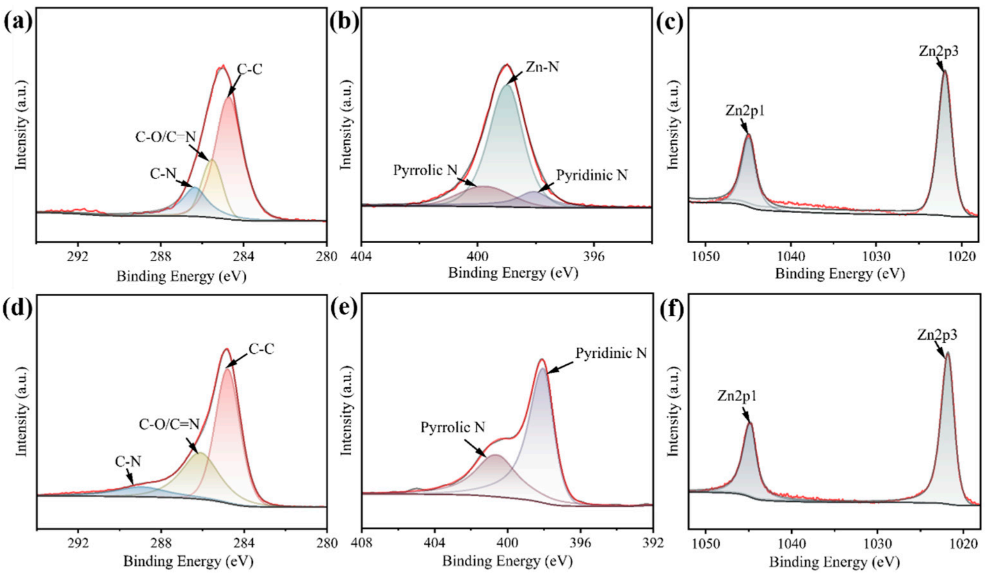

Figure S16 XPS spectra of ZIF-8: (a) C 1s, (b) N 1s, and (c) Zn 2p; XPS spectra of G@ZIF-8: (d) C 1s, (e) N 1s, and (f) Zn 2p.

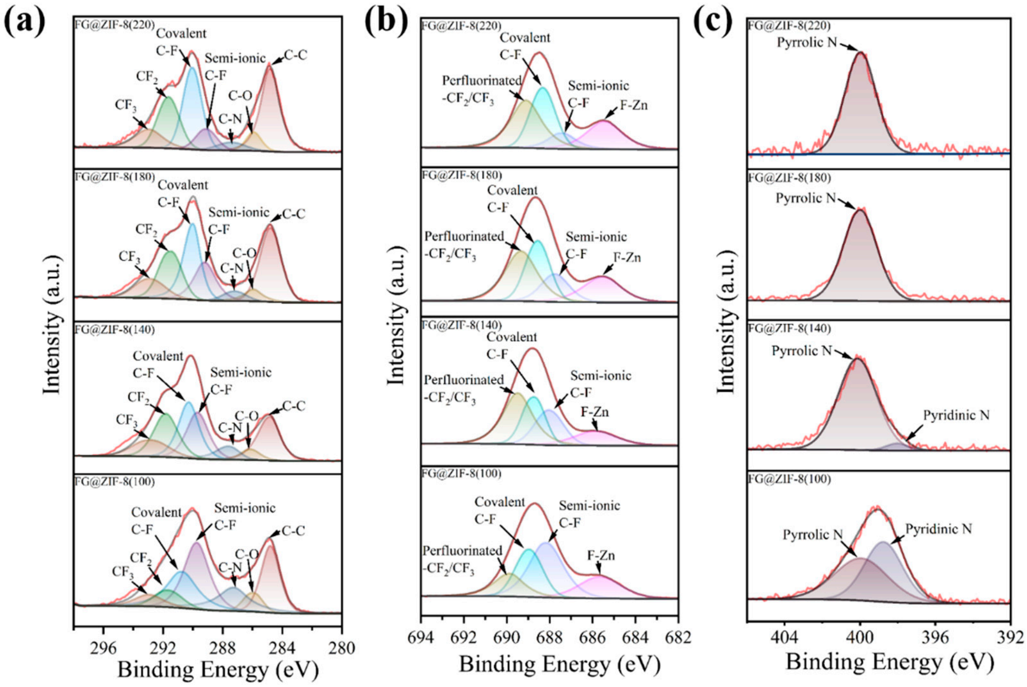

Figure S17 XPS spectra of FG@ZIF-8 prepared at different temperatures: (a) C 1s, (b) F 1s, and (c) N 1s.

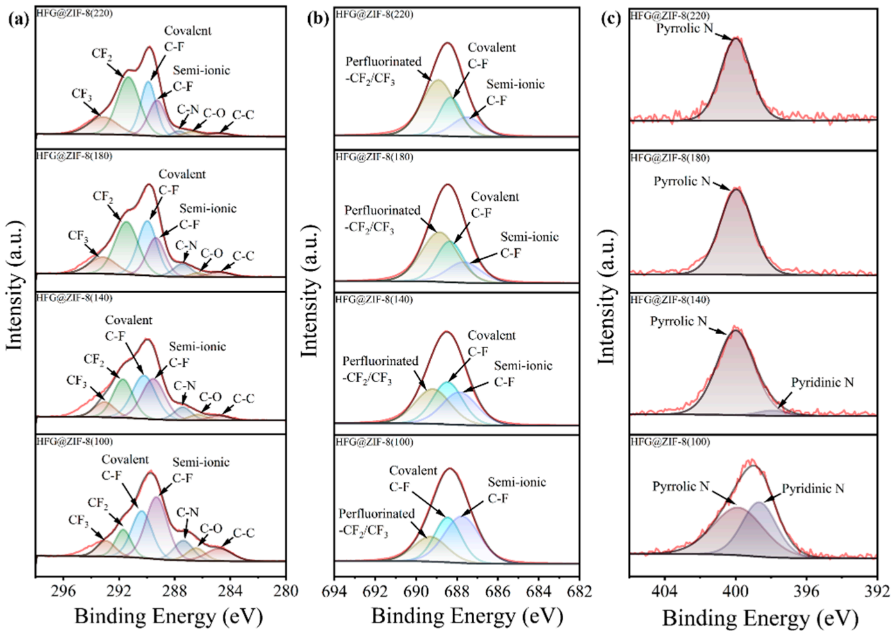

Figure S18 XPS spectra of HFG@ZIF-8 prepared at different temperatures: (a) C 1s, (b) F 1s, and (c) N 1s.

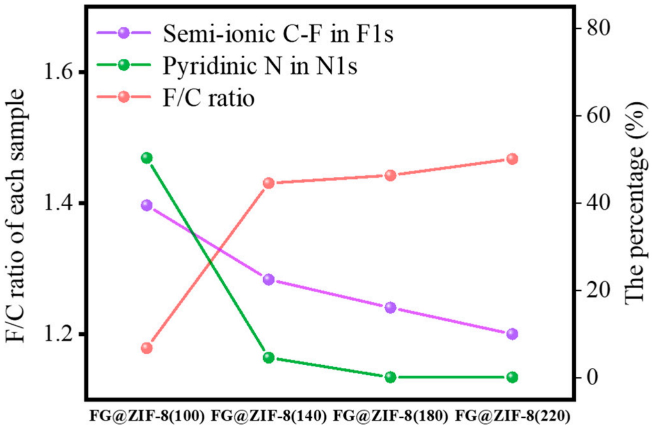

Figure S19 F/C ratio, semi-ionic C–F content in F 1s, and pyridinic N content in N 1s as a function of fluorination temperature for FG@ZIF-8.

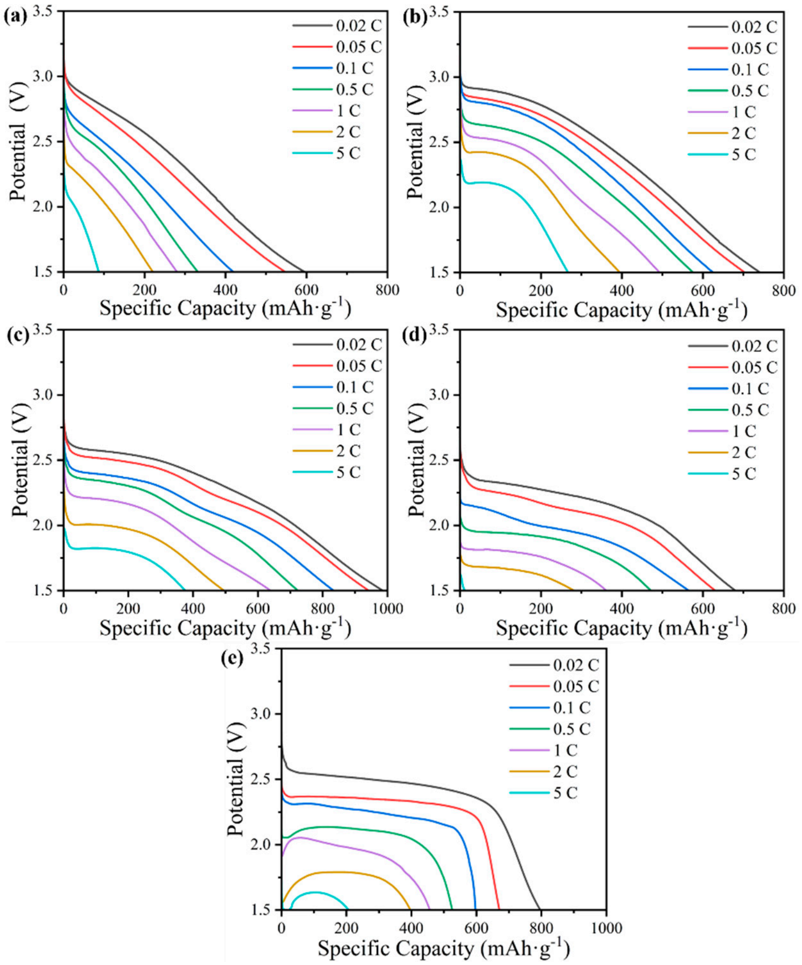

Figure S20 Galvanostatic discharge curves of (a) FG@ZIF-8(100), (b) FG@ZIF-8(140), (c) FG@ZIF-8(180), (d) FG@ZIF-8(220), and (e) GF.

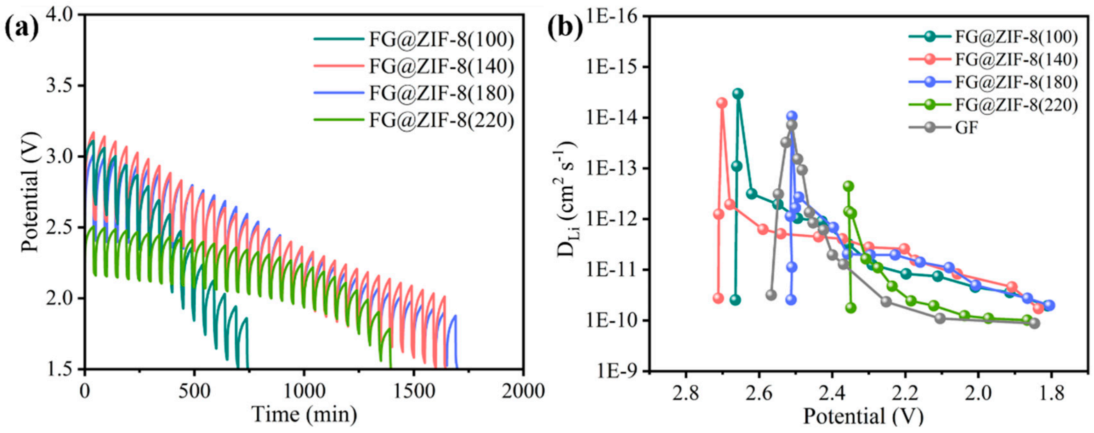

Figure S21 (a) GITT curves of FG@ZIF-8 at 0.1 C, and (b) relationship between D_{Li^+} and voltage for FG@ZIF-8.

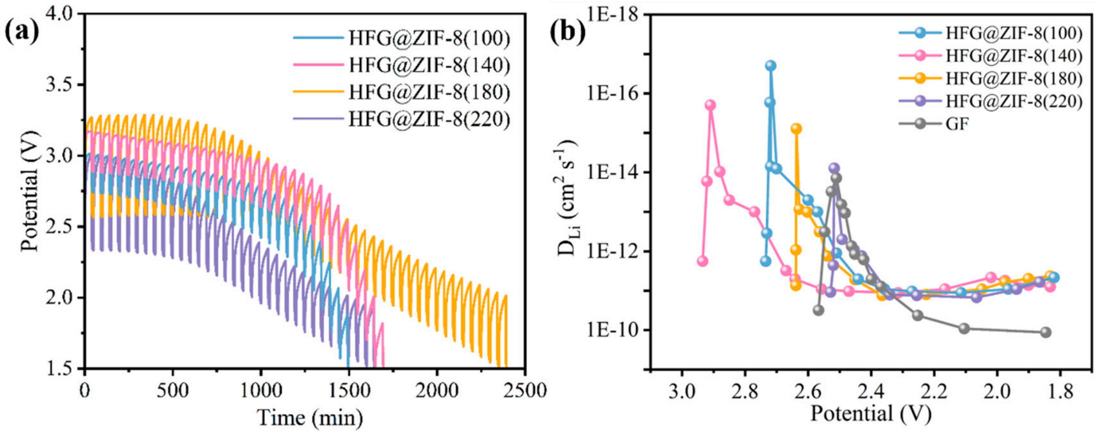

Figure S22 (a) GITT curves of HFG@ZIF-8 at 0.1 C, and (b) relationship between D_{Li^+} and voltage for HFG@ZIF-8.

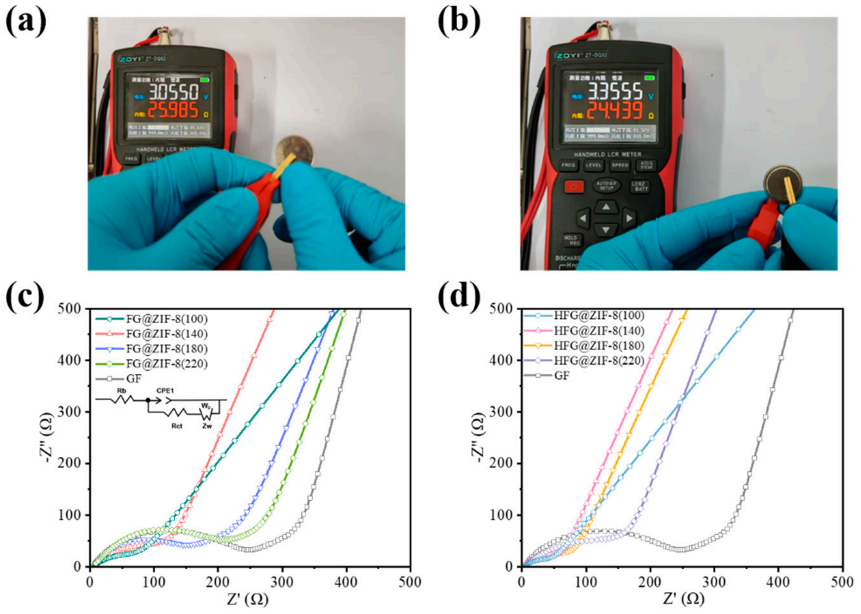

Figure S23 The open-circle voltage of FG@ZIF-8 (a) and HFG@ZIF-8 (b), EIS spectra of FG@ZIF-8 (c) and HFG@ZIF-8 (d).

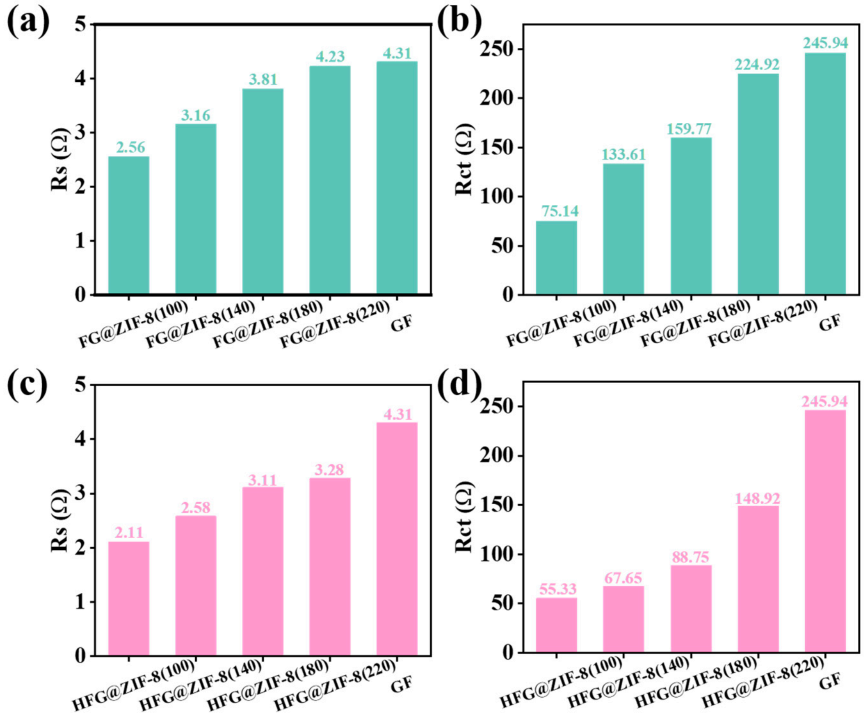

Figure S24 Bulk ohmic resistance (R_s) and charge-transfer resistance (R_{ct}) of FG@ZIF-8 (a, b) and HFG@ZIF-8 (c, d).

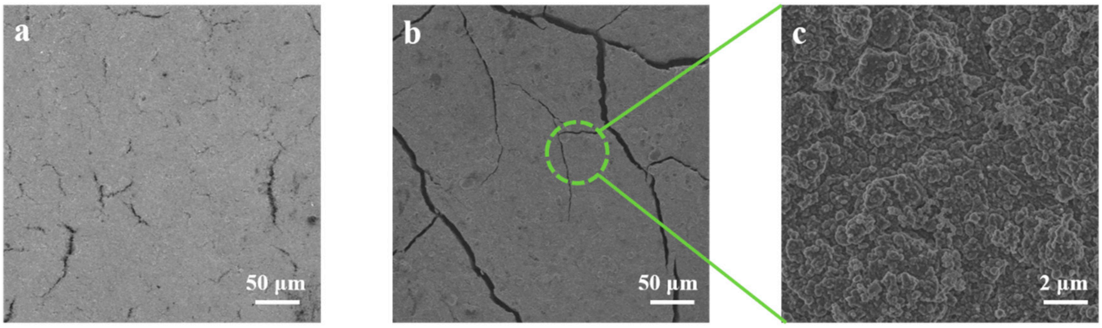

Figure S25 SEM images of the HFG@ZIF-8(180) cathode before discharge (a) and after discharge (b–c).

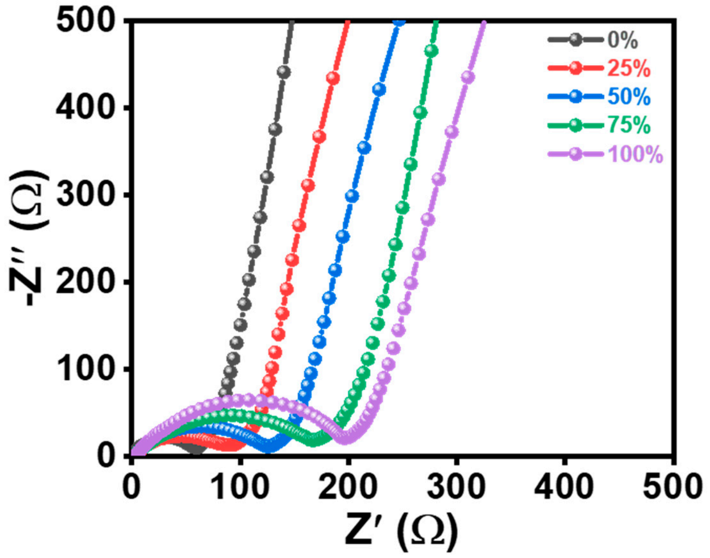

Figure S26 EIS spectra of HFG@ZIF-8(180) at different depth of discharge.

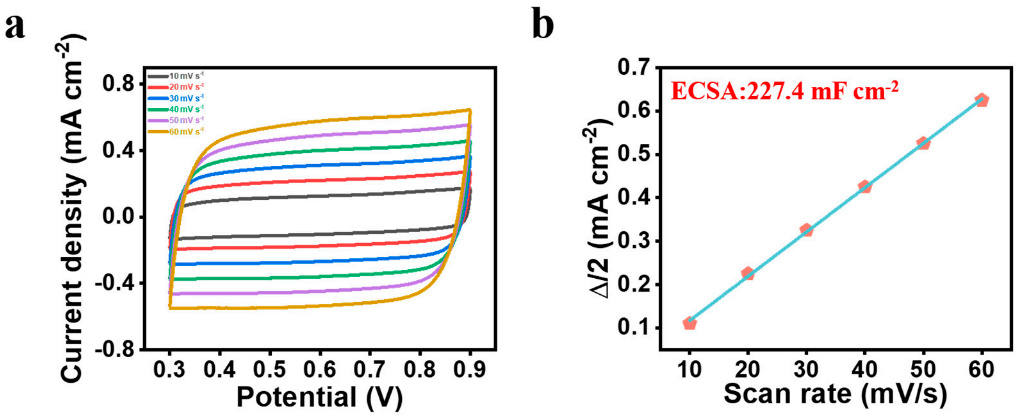

Figure S27 (a) CV curves of HFG@ZIF-8(180) at different scan rates, (b) Linear plots of scan rates versus current density of HFG@ZIF-8(180).

Calculation of Effective Active Surface Area (ECSA): the double layer capacitance (C_{dl}) is obtained by cyclic voltammetry at different scan rates (in the range of 10~60 mV s^{-1}) to be linearly proportional to effective active surface area (ECSA). The potential is in the range from 0.3 to 0.9 V, the C_{dl} is estimated by plotting the difference between anodic and cathodic currents

(j_a-j_c) at 0.6 V against various scan rates, where the slope is double C_{dl}. The specific capacitance C_{dl} can be converted into an electrochemical active surface area (ECSA) by the following equation, and The specific capacitance for a flat surface is generally found to be 40 μF cm^{-2} [S1].

$$ECSA = {}^{C_{dl}}/_{C_s}$$

S1. Li, Y.; Yu, X.; Liu, Y.; Liu, C.; Jin, J.; Hao, L.; Zhang, T.; Tang, X.; Xiong, P.; Zhu, J. Regulating Spin Configuration of Mn Single Atoms/Mn Atomic Clusters Catalysts for High-Performance Zinc-Air Batteries. Nano Energy 2025, 146, 111498, doi:10.1016/j.nanoen.2025.111498.

Table S1 BET surface area and pore volume of ZIF-8, G@ZIF-8, and FG@ZIF-8.

Sample	S_{BET}(m^2/g)	V_{tot} (cm^3/g)
ZIF-8	1678.7	0.714
G@ZIF-8	200	0.456
FG@ZIF-8(100)	323.9	0.573
FG@ZIF-8(140)	194.3	0.405
FG@ZIF-8(180)	171.2	0.398
FG@ZIF-8(220)	5.2	0.041

Table S2 BET surface area and pore volume of HFG@ZIF-8.

Sample	S_{BET}(m^2/g)	V_{tot} (cm^3/g)
HFG@ZIF-8(100)	266.7	0.526
HFG@ZIF-8(140)	215.5	0.417
HFG@ZIF-8(180)	207.5	0.405
HFG@ZIF-8(220)	179.3	0.301

Table S3 The interlayer space of HFG@ZIF-8 (obtained from XRD results).

Sample	2θ (°)	The interlayer space (nm) $d_{(001)}$
HFG@ZIF-8(100)	9.7	0.91
HFG@ZIF-8(140)	12.1	0.731
HFG@ZIF-8(180)	12.48	0.708
HFG@ZIF-8(220)	13.54	0.657

Table S4 Composition contents of ZIF-8, G@ZIF-8, FG@ZIF-8 from the XPS spectra.

Sample	C (At. %)	N (At. %)	O (At. %)	F (At. %)	Zn (At. %)
ZIF-8	59.9	23.08	5.98	1.67	9.37
G@ZIF-8	65.81	13.24	16.61	1.21	3.13
FG@ZIF-8(100)	38.83	6.1	6.08	45.73	3.25
FG@ZIF-8(140)	36.8	4.19	3.05	52.62	3.33
FG@ZIF-8(180)	36.16	3.09	3.18	52.16	5.42
FG@ZIF-8(220)	35.83	2.63	3.1	52.57	5.87

Table S5 Composition contents of HFG@ZIF-8 from the XPS spectra.

Sample	C (At. %)	N (At. %)	O (At. %)	F (At. %)	Zn (At. %)
HFG@ZIF-8(100)	39.51	6.68	5.42	48.2	0.19
HFG@ZIF-8(140)	35.71	5.96	1.87	56.3	0.15
HFG@ZIF-8(180)	35.68	4.62	1.89	57.71	0.1
HFG@ZIF-8(220)	35.58	3.46	1.34	59.45	0.17

Table S6 C 1s peak assignments and relative proportions of ZIF-8, G@ZIF-8, and FG@ZIF-8 derived from XPS spectra.

Sample	C1s assignment and FWHM (eV)							
	C-C (%)	C-N (%)	C=N (%)	C-O (%)	Semi-ionic C-F (%)	Covalent C-F (%)	CF_2 (%)	CF_3 (%)
ZIF-8	284.8 eV<br>(59.5%)<br>2.01 eV	286.4 eV<br>(17.5%)<br>1.37 eV	285.5 eV<br>(23%)<br>1.55 eV		/	/	/	/
G@ZIF-8	284.8 eV<br>(56.7%)<br>2.07 eV	288.9 eV<br>(10.9%)<br>1.41 eV	286.1 eV<br>(32.4%)<br>1.52 eV		/	/	/	/
FG@ZIF-8(100)	284.8 eV<br>(20%)<br>2.01 eV	284.8 eV<br>(59.5%)<br>2.04 eV	/	285.9 eV<br>(5.6%)<br>2.04 eV	289.7 eV<br>(27.4%)<br>2.11 eV	290.7 eV<br>(19.6%)<br>2.47 eV	291.7 eV<br>(7.3%)<br>1.98 eV	293.0 eV<br>(5.5%)<br>1.81 eV
FG@ZIF-8(140)	284.8 eV<br>(18.8%)<br>2.02 eV	284.8 eV<br>(59.5%)<br>2.09 eV	/	286.2 eV<br>(3.8%)<br>2.07 eV	289.7 eV<br>(19.0%)<br>2.10 eV	290.3 eV<br>(21.6%)<br>2.51 eV	291.8 eV<br>(19.3%)<br>2.04 eV	292.8 eV<br>(10.6%)<br>1.92 eV
FG@ZIF-8(180)	284.8 eV<br>(26.1%)<br>2.05 eV	284.8 eV<br>(59.5%)<br>2.1 eV	/	286.0 eV<br>(4.2%)<br>2.06 eV	289.2 eV<br>(14.5%)<br>2.14 eV	290.0 eV<br>(23.6%)<br>2.52 eV	291.5 eV<br>(18.2%)<br>2.04 eV	292.9 eV<br>(9.7%)<br>1.99 eV
FG@ZIF-8(220)	284.8 eV<br>(29.4%)<br>2.05 eV	284.8 eV<br>(59.5%)<br>2.11 eV	/	285.9 eV<br>(5.0%)<br>2.08 eV	289.1 eV<br>(7.4%)<br>2.15 eV	290.0 eV<br>(26.0%)<br>2.55 eV	291.6 eV<br>(19.2%)<br>2.04 eV	293.0 eV<br>(9.1%)<br>2.00 eV

Table S7 F 1s peak assignments and relative proportions of FG@ZIF-8 derived from XPS spectra.

Sample	F1s assignment (eV)							
	Zn-F (%)	FWHM (eV)	Semi-ionic C-F (%)	FWHM (eV)	Covalent C-F (%)	FWHM (eV)	CF_2/CF_3 (%)	FWHM (eV)
FG@ZIF-8(100)	685.7 eV (18.8%)	2.12	688.2 eV (39.5%)	1.99	689.0 eV (26.1%)	2.42	689.9 eV (15.6%)	2.07
FG@ZIF-8(140)	685.9 eV (14.7%)	2.05	688.0 eV (22.4%)	2.05	688.7 eV (25.1%)	2.51	689.5 eV (37.8%)	2.14
FG@ZIF-8(180)	685.5 eV (20.6%)	2.08	687.7 eV (16.0%)	2.11	688.6 eV (29.4%)	2.5	689.3 eV (34%)	2.17
FG@ZIF-8(220)	685.5 eV (22.5%)	2.17	687.4 eV (9.9%)	2.09	688.3 eV (32.5%)	2.56	688.1 eV (35.1%)	2.11

Table S8 C 1s peak assignments and relative proportions of HFG@ZIF-8 derived from XPS spectra.

Sample	C1s assignment and FWHM (eV)						
	C-C (%)	C-N (%)	C-O (%)	Semi-ionic C-F (%)	Covalent C-F (%)	CF_2 (%)	CF_3 (%)
HFG@ZIF-8(100)	284.8 eV (8.3%) 1.98 eV	287.4 eV (9.6%) 2.00 eV	286.5 eV (5.8%) 2.03 eV	289.3 eV (34.0%) 2.10 eV	290.4 eV (24.3%) 2.54 eV	291.7 eV (10.8%) 1.99 eV	292.9 eV (7.2%) 1.98 eV
HFG@ZIF-8(140)	284.8 eV (2.9%) 1.97 eV	287.4 eV (6.4%) 1.89 eV	286.3 eV (2.8%) 1.98 eV	289.5 eV (27.1%) 2.09 eV	290.2 eV (29.5%) 2.55 eV	291.7 eV (22.3%) 1.98 eV	293.0 eV (9.0%) 1.92eV
HFG@ZIF-8(180)	284.8 eV (2.9%) 1.98 eV	287.4 eV (6.1%) 1.92 eV	286.3 eV (1.5%) 1.99 eV	289.4 eV (18.1%) 2.11 eV	290.0 eV (27.2%) 2.49 eV	291.5 eV (32.4%) 1.98 eV	293.1 eV (11.8%) 1.90 eV
HFG@ZIF-8(220)	284.8 eV (2.3%) 2.00 eV	287.7 eV (2.0%) 2.01 eV	286.7 eV (2.5%) 1.95 eV	289.3 eV (17.8%) 2.12 eV	289.9 eV (23.8%) 2.61 eV	291.3 eV (37.2%) 2.04 eV	293.1 eV (14.4%) 2.05 eV

Table S9 F 1s peak assignments and relative proportions of HFG@ZIF-8 derived from XPS spectra.

Sample	F1s assignment (eV)					
	Semi-ionic C-F (%)	FWHM (eV)	Covalent C-F (%)	FWHM (eV)	CF_2/CF_3 (%)	FWHM (eV)
HFG@ZIF-8(100)	687.8 eV (44.9%)	2.00	688.5 eV (32.5%)	2.44	689.3eV (22.6%)	2.07
HFG@ZIF-8(140)	687.9 eV (31.4%)	2.07	688.5 eV (34.8%)	2.48	689.2 eV (33.8%)	2.06
HFG@ZIF-8(180)	687.7 eV (19.4%)	2.05	688.4 eV (31.0%)	2.57	687.3 eV (49.6%)	2.04
HFG@ZIF-8(220)	687.5 eV (17.5%)	1.97	688.3 eV (26.7%)	2.52	688.9 eV (55.8%)	2.01
